# Cultural adaptation, validation, and first application of the Canadian assessment of physical literacy (CAPL-2) in Lithuanian primary school children

**DOI:** 10.3389/fspor.2026.1922697

**Published:** 2026-08-19

**Authors:** Audrone Vizbariene, Brigita Mieziene, Renata Rutkauskaite, Arunas Emeljanovas

**Affiliations:** Department of Physical and Social Education, Lithuanian Sports University, Kaunas, Lithuania

**Keywords:** CAPL-2, cross-cultural adaptation and validation, physical education, physical literacy, primary school children

## Abstract

**Introduction:**

Physical literacy is increasingly recognized as a multidimensional construct that supports lifelong engagement in physical activity. Although the Canadian Assessment of Physical Literacy, second edition (CAPL-2), is one of the most widely used instruments for assessing physical literacy in children, no culturally adapted and validated Lithuanian version has previously been available. This study aimed to translate and culturally adapt CAPL-2 for Lithuanian primary school children, evaluate its psychometric properties, and report the findings from its first application in a national sample.

**Methods:**

CAPL-2 was translated and culturally adapted following established international guidelines, including forward–backward translation, expert review, and pilot testing. The adapted instrument was administered to 846 children aged 8–11 years from 10 Lithuanian schools. Descriptive statistics, Pearson correlations, confirmatory factor analysis, age- and sex-group comparisons, and internal consistency analyses were performed.

**Results:**

The Lithuanian CAPL-2 demonstrated good psychometric properties and retained the original four-domain structure (CFI = .963, TLI = .946, RMSEA = .047, SRMR = .040). The Motivation and Confidence domain showed good internal consistency (Cronbach's *α* = .867; McDonald's *ω* = .87). The mean total CAPL-2 score was 70.25/100. Motivation and Confidence showed the highest domain scores, whereas Knowledge and Understanding demonstrated the weakest associations with the remaining domains and no significant correlation with Daily Behavior. Significant age-related differences were observed for Physical Competence and Knowledge and Understanding.

**Conclusion:**

The Lithuanian version of CAPL-2 is a valid and culturally appropriate instrument for assessing physical literacy in primary school children. The findings support its use in research, education, and public health and provide a foundation for future national and international studies.

## 1 Introduction

Research indicates that children's physical activity (PA) begins to decline from the early school years (around ages 7–9) and continues to decrease gradually over time, with similar trends observed among both boys and girls worldwide (1–3). Early declines in PA, together with increasing sedentary behavior, have important health implications, including an increased risk of obesity, cardiovascular disease, and psychosocial problems (1, 4–6). Moreover, children who are least physically active during primary school are substantially more likely to remain physically inactive in young adulthood (7).

Primary school years are considered a particularly important stage for implementing interventions to promote PA. However, children's PA is influenced by a complex interplay of psychosocial, individual, and environmental factors, including motivation, self-efficacy, screen time, physical fitness, body mass index, academic demands, and opportunities for PA (1, 4, 8–11). These findings suggest the need for holistic approaches that focus not only on increasing PA itself but also on developing the competencies that support sustained engagement in an active lifestyle. In this context, physical literacy (PL) has emerged as an increasingly important concept. PL is most often defined as a multidimensional construct encompassing motivation, confidence, physical competence, knowledge, and understanding, which together support lifelong engagement in PA (12–14). Higher levels of PL have consistently been associated with greater PA, lower sedentary behavior, and healthier movement patterns in children (15–18).

As the importance of PL grows, its valid assessment is becoming one of the most critical areas of research, education, and health promotion. This is particularly evident in Europe, where interest in PL has increased considerably in recent years, yet its implementation and assessment remain heterogeneous across countries (19). Reliable assessment methods are essential for monitoring children's development, evaluating the effectiveness of interventions, improving pedagogical practice, and developing evidence-based policies (20–22). However, the assessment of PL remains challenging due to the lack of a universally accepted definition and the difficulties in capturing its multidimensional and holistic nature (22).

Among the available instruments, the Canadian Assessment of Physical Literacy, second edition (CAPL-2), is one of the most widely used and comprehensive tools for assessing PL in children aged 8–12 years. CAPL-2 is based on the internationally accepted definition of PL adopted by the International Physical Literacy Association, which defines PL as motivation, confidence, physical competence, knowledge, and understanding to value and take responsibility for engagement in physical activities for life (23). Accordingly, CAPL-2 operationalizes this definition by assessing four complementary domains: Physical Competence, Daily Behavior, Motivation and Confidence, and Knowledge and Understanding, by using combination of objective physical tests and self-report questionnaires (23, 24). The instrument has demonstrated strong psychometric properties and has been widely validated across various populations (22, 25). Furthermore, the CAPL-2 has been translated and culturally adapted across multiple countries, including European and Asian contexts (26–34), consistently demonstrating acceptable validity and reliability, although some variability across domains has been reported (26–28). In some countries, these differences have led to modifications of specific indicators or model parameters (27, 28), highlighting the importance of evaluating the instrument within each cultural context.

Owing to its multidimensional structure, strong psychometric properties, and applicability in school settings, CAPL-2 is currently considered one of the reference standards for assessing PL in children aged 8–12 years (23, 25). Its validation is particularly relevant in Lithuania, where the national primary physical education curriculum emphasizes holistic child development, lifelong engagement in physical activity, self-assessment, and personal progress rather than physical performance alone (35). Despite this educational orientation, research on PL in Lithuania is still at an early stage (19) and has primarily focused on conceptual discussions, with only limited empirical evidence available regarding the comprehensive assessment of PL (36). To date, only one pilot study has applied a modified version of CAPL in Lithuanian primary school children, and its authors emphasized the need for cultural adaptation and validation of the instrument for the Lithuanian population (37). Consequently, no culturally adapted and validated Lithuanian version of CAPL-2 is currently available for comprehensively assessing PL among Lithuanian primary school children. To address this gap, the aim of the present study was to translate and culturally adapt the CAPL-2 for Lithuanian primary school children, evaluate its psychometric properties, and report the findings from its first application in a national sample.

## 2 Materials and methods

### 2.1 Study design

This study employed a quantitative cross-sectional design to culturally adapt and validate the Lithuanian version of CAPL-2 and to examine its first application among Lithuanian primary school children. The study was grounded in a holistic conceptualization of PL, where Physical Competence, Motivation and Confidence, Knowledge and Understanding, and Daily Behavior are considered interrelated domains supporting engagement in PA (13, 23). The study included the translation and cultural adaptation of the instrument, assessment of its psychometric properties, and description of PL characteristics in a national sample of Lithuanian primary school children.

### 2.2 Participants and sampling

Participants were selected using a cluster probability sampling approach, in which general education schools providing primary education served as the sampling units. The national Register of Educational and Science Institutions (38) was used to identify eligible schools. The study was conducted across all ten counties of Lithuania to ensure geographic coverage and capture the diversity of the national population of primary school children. In each county, one urban, small-town, or rural location was randomly selected, and within each location, one general education primary school was invited to participate, considering the logistical feasibility of data collection yielding a total of 10 schools, all of which agreed to participate.

All students in Grades 2–4 from the selected schools were invited to participate. The study included only those children whose parents or legal guardians provided written informed consent. Participating children were also provided with an age-appropriate verbal explanation of the study and informed that participation was voluntary and that they could withdraw at any time without any consequences.

**Figure 1:**
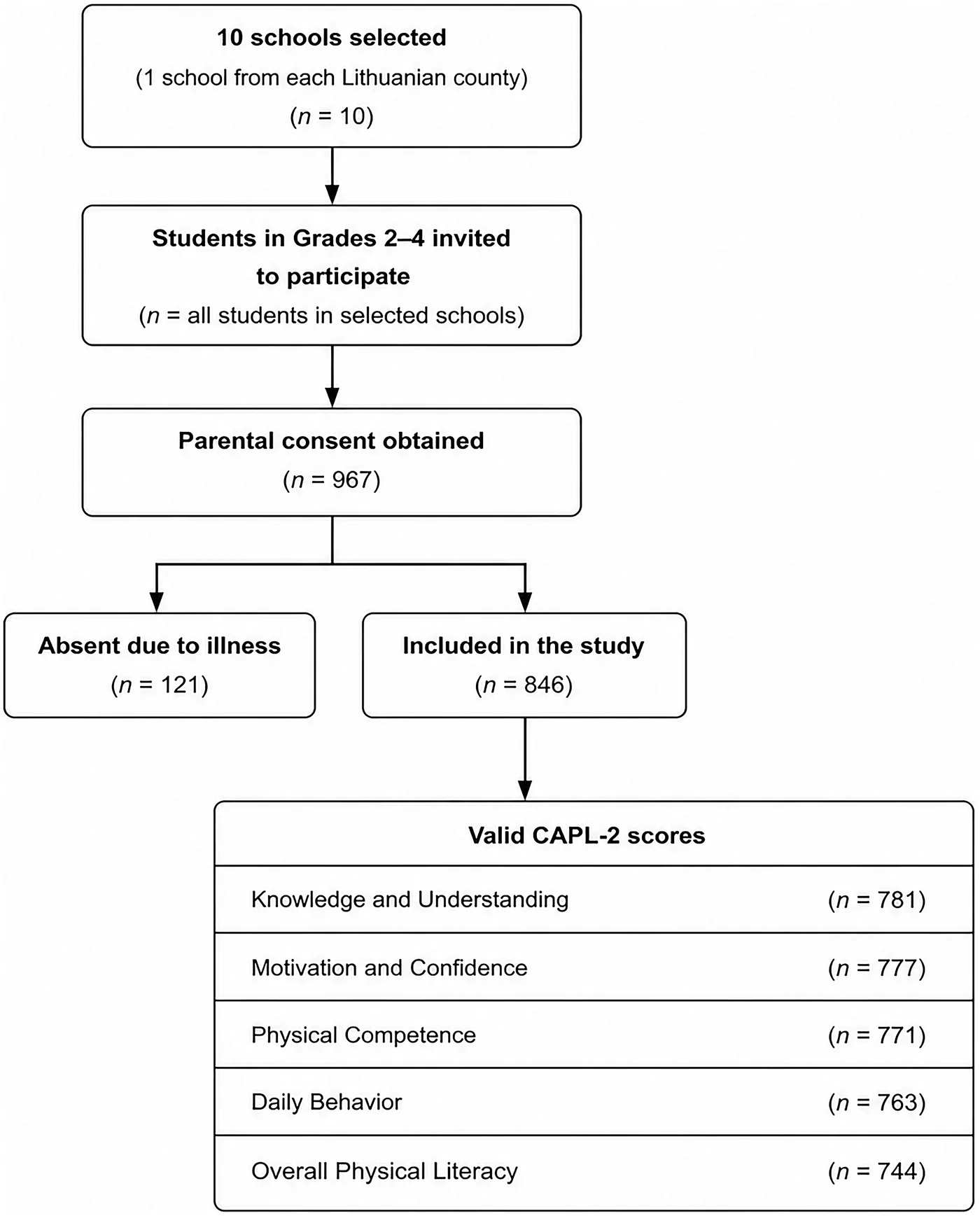
Flow diagram of participant recruitment and final study sample.

Following the recruitment process, 967 parental informed consent forms were obtained. However, the final study sample consisted of 846 primary school children (87.5% of those with parental consent), including 421 boys (49.8%) and 425 girls (50.2%). Participants ranged in age from 8 to 11 years. As no universally accepted sample size recommendations exist for instrument validation studies (39), the adequacy of the achieved sample was evaluated using two complementary approaches. The study sample was comparable to or larger than the sample sizes reported in previous national CAPL-2 validation studies (26, 27, 32). In addition, because confirmatory factor analysis (CFA) was one of the primary psychometric analyses planned in this study, sample adequacy was also evaluated using the participant-to-parameter ratio. With 846 participants and 40 estimated parameters, the ratio was approximately 21:1, exceeding commonly recommended thresholds for stable parameter estimation in structural equation modeling (40, 41)**.** A total of 121 students were absent from school due to illness at the beginning of the data collection process and were therefore not included in the study (Figure 1).

Due to missing data across individual CAPL-2 assessment protocols, the number of valid observations varied across analyses. Valid scores were available for 781 participants in Knowledge and Understanding, 777 in Motivation and Confidence, 771 in Physical Competence, 763 in Daily Behavior, and 744 for the Overall Physical Literacy score, which was calculated in accordance with the CAPL-2 scoring procedure. Participants with more than one missing assessment protocol were not assigned an Overall Physical Literacy score (42)

### 2.3 Translation and cultural adaptation of the CAPL-2 instrument

As CAPL-2 had not previously been adapted for use in the Lithuanian context, a systematic process of translation and cultural adaptation was conducted prior to the empirical study. The translation and adaptation process followed established international guidelines for the translation and cultural adaptation of measurement instruments (43) and aimed to ensure semantic, conceptual, and content equivalence between the original and Lithuanian versions.

First, two independent bilingual translators, whose native language was Lithuanian and who had experience in PA and education-related fields, independently translated the original CAPL-2 questionnaire and physical test protocols into Lithuanian. The two translated versions were subsequently reviewed and synthesized by a third bilingual researcher experienced in the use of assessment instruments, resulting in a preliminary Lithuanian version.

The preliminary version was then back-translated into English by an independent translator whose native language was English and who was blinded to the original CAPL-2 instrument. The back-translated version was compared with the original instrument to identify potential semantic and conceptual discrepancies. Subsequently, the Lithuanian version was evaluated by an expert committee consisting of a physical education researcher, a primary school teacher, and the translators involved in the adaptation process. The committee assessed item clarity, appropriateness of terminology, cultural relevance, and suitability for the target age group, and all identified issues were discussed and resolved through consensus.

Finally, the revised Lithuanian version was pilot tested with six primary school children (two second graders, two third graders, and two fourth graders) representing the target age range. Children completed the questionnaire independently while researchers observed the process and recorded any difficulties related to item comprehension, instructions, or wording. Brief cognitive debriefing interviews were subsequently conducted to obtain feedback on question clarity and comprehensibility. Minor linguistic adjustments were made based on the children's feedback, and the final version was considered suitable for use in the main study.

### 2.4 Assessment of PL using CAPL-2

PL was assessed using CAPL-2, a multidimensional instrument designed to evaluate children's engagement in PA across behavioral, physical, cognitive, and affective domains (23). Administration procedures and scoring protocols followed the official CAPL-2 training and implementation materials provided by the CAPL research team (42). Motivation and Confidence were assessed using a 12-item self-report questionnaire encompassing four constructs: Predilection, Adequacy, Intrinsic Motivation, and Perceived Competence. Responses were scored according to CAPL-2 guidelines, yielding a maximum of 30 points, with higher scores indicating stronger motivation and confidence toward PA.

Physical Competence was evaluated using three performance-based measures. Aerobic fitness was assessed using the 20 m Progressive Aerobic Cardiovascular Endurance Run (PACER), with the number of completed laps recorded. Muscular endurance was measured using an isometric plank hold, with time recorded in seconds. Motor skill proficiency and agility were assessed using the Canadian Agility and Movement Skill Assessment (CAMSA), which integrates locomotor and object control skills within a timed obstacle course.

Each test was scored on a scale from 0 to 10 points according to CAPL-2 scoring procedures, and the combined score for the Physical Competence domain ranged from 0 to 30 points.

Knowledge and Understanding were assessed using a five-item questionnaire addressing key concepts related to PA and fitness, including recommended activity levels and basic fitness principles. Responses were scored according to standardized criteria, with a maximum of 10 points.

Daily Behavior was assessed using both objective and self-reported measures. Objective PA was recorded using a wrist-worn accelerometer (ActiGraph wGT3X-BT) worn on the non-dominant wrist for seven consecutive days. Although the original CAPL-2 protocol recommends a waist-worn pedometer, a wrist-worn accelerometer was used because this approach has been shown to provide high wear compliance in children during prolonged monitoring (44). Daily step counts were obtained directly from the ActiLife 6.13.4 Daily Summary output using the proprietary step-detection algorithm implemented in the software. No additional calibration or adjustment of step counts was performed prior to applying the standard CAPL-2 scoring procedure. The recorded daily step counts were then converted into CAPL-2 Daily Behavior scores according to the standard CAPL-2 scoring guidelines. Participants with at least four valid days of monitoring were included in the calculation of average daily step counts. When only three valid days were available, missing data were handled according to CAPL-2 procedures (42). Average daily step counts were converted into CAPL-2 scores of up to 25 points. Self-reported PA was assessed by asking participants to report the number of days in the past week during which they accumulated at least 60 min of moderate-to-vigorous PA. This component contributed up to 5 additional points. The total PL score was calculated by summing all domain scores, with a maximum of 100 points.

Data collection was conducted in two sessions separated by a one-week interval. During the first session, participants were provided with wrist-worn accelerometers and instructed to wear them on the non-dominant hand for seven consecutive days. On the same day, Physical Competence was assessed using the plank and Canadian Agility and Movement Skill Assessment (CAMSA) tests. During the second session, participants completed the Progressive Aerobic Cardiovascular Endurance Run (PACER) test and the CAPL-2 questionnaire components. At the end of the session, accelerometers were collected. All assessments were conducted by the same research team with the assistance of trained volunteer physical education teachers, following standardized procedures.

### 2.5 Ethical considerations

The study was conducted in accordance with established ethical principles for research involving human participants. Ethical approval was obtained from the Social Sciences Research Ethics Committee of Lithuanian Sports University in 2024 (Protocol No. SMTEK-272).

Participation was voluntary, and only children whose parents or legal guardians provided informed consent through an electronic consent form were included in the study. Participants and their parents or guardians were informed that participation was voluntary and that withdrawal from the study was possible at any time without consequences. All collected data were coded and anonymized to ensure participant confidentiality.

### 2.6 Data analysis

Descriptive statistics (means, standard deviations, ranges, skewness, and kurtosis) were calculated to summarize the characteristics of the study variables. Pearson correlation coefficients were used to examine relationships among CAPL-2 domains and the total PL score. Known-groups validity, as one aspect of construct validity, was examined by comparing CAPL-2 scores across age and sex groups**.** Differences across age groups were examined separately for boys and girls using one-way analysis of variance (ANOVA). When significant overall effects were identified, Bonferroni *post hoc* tests were performed to determine pairwise differences between age groups. Sex differences within each age group were examined using independent-samples t-tests.

Internal consistency of the Motivation and Confidence domain, which comprises multiple questionnaire items, was assessed using Cronbach's alpha and McDonald's omega coefficients. Values ≥ 0.70 were considered indicative of acceptable internal consistency.

Construct validity of the Lithuanian version of CAPL-2 was evaluated using confirmatory factor analysis (CFA). The analysis was conducted in R (version 4.5.3) using the *lavaan* package with maximum likelihood estimation and full information maximum likelihood (FIML) to handle missing data. Model fit was assessed using multiple indices, including the Comparative Fit Index (CFI), Tucker–Lewis Index (TLI), Root Mean Square Error of Approximation (RMSEA), and Standardized Root Mean Square Residual (SRMR). Acceptable model fit was defined as CFI and TLI ≥ 0.90, RMSEA ≤ 0.06, and SRMR ≤ 0.08.

Model refinement was limited and guided by theoretical considerations and previous research. Specifically, a correlation between the error terms of the Predilection and Adequacy indicators was included, consistent with prior findings (Elsborg et al., 2021). McDonald's omega was calculated in R (version 4.5.3), whereas all other statistical analyses were performed using IBM SPSS Statistics (Version 28). Statistical significance was set at *p* < .05.

## 3 Results

### 3.1 Descriptive statistics and correlations among CAPL-2 domains

Results are based on data collected from 846 primary school children (421 boys and 425 girls). Due to the requirement for the complete data for each CAPL-2 assessment component, the number of valid observations varied across CAPL-2 components, ranging from 744 to 781 participants.

Descriptive statistics and correlations among the four CAPL-2 domains and the total CAPL-2 score are presented in Table 1.

**Table 1:** Descriptive statistics and correlations among the four domains and total CAPL-2 score (Lithuanian version).

		Descriptives							Correlation			
Variable (score range)	N	Mean	SD	min	max	% of max	skew	kurt	1	2	3	4
1. Total CAPL-2 Score	744	70.25	12.10	30.50	98.60	70.3	-.37	-.22	1.00			
2. Motivation and Confidence	777	25.71	4.00	10.90	30.00	85.7	-1.00	.53	.65**	1.00		
3. Daily Behavior	763	19.81	6.33	1.00	30.00	66.0	-.62	-.26	.70**	.21**	1.00	
4. Physical Competence	771	19.38	5.59	2.43	30.00	64.6	-.08	-.61	.77**	.41**	.24**	1.00
5. Knowledge and Understanding	781	5.33	2.27	.00	10.00	53.3	-.07	-.65	.34**	.12**	-.01	.24**

**p < .01 (two-tailed).

The mean CAPL-2 total score was 70.25 out of a possible 100 points. Among the four domains, expressed as a percentage of the maximum possible domain score, Motivation and Confidence was highest (85.7%), followed by Daily Behavior (66.0%) and Physical Competence (64.6%), whereas Knowledge and Understanding had the lowest score (53.3%). Skewness values ranged from −1.00 to −0.07 and kurtosis values ranged from −0.65 to 0.53 (Table 1), indicating no substantial deviations from normality. All CAPL-2 domains were positively correlated with the total CAPL-2 score. Physical Competence demonstrated the strongest association with the total score (*r* = .77, *p* < .01), while Knowledge and Understanding showed the weakest (*r* = .34, *p* < .01). Positive correlations were also observed among most CAPL-2 domains. However, no significant correlation was found between Daily Behavior and Knowledge and Understanding (*r* = −.01, *p* > .05).

### 3.2 Confirmatory factor analysis of the Lithuanian version of CAPL-2

**Figure 2:**
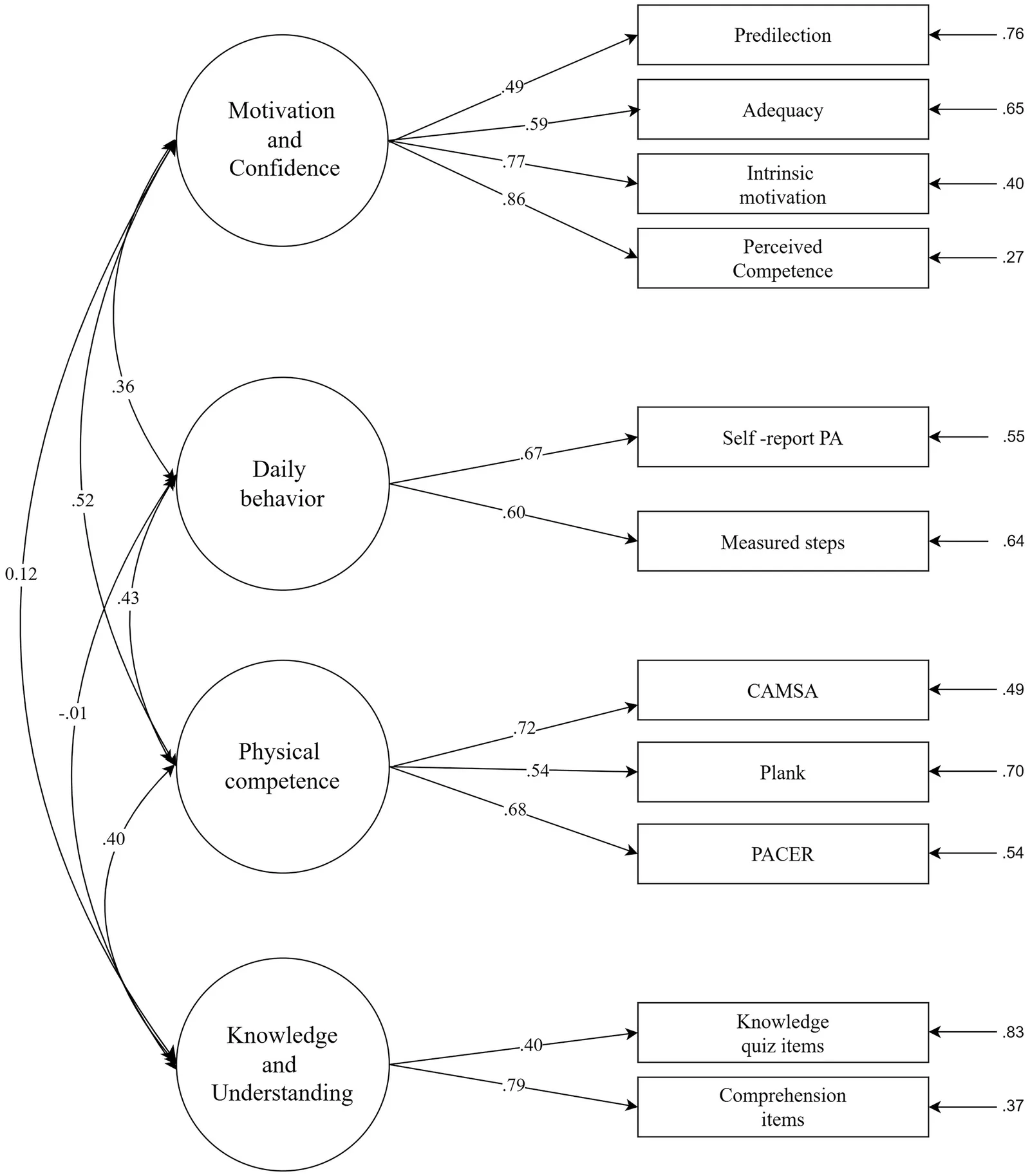
Confirmatory factor analysis of the Lithuanian version of CAPL-2.

Confirmatory factor analysis was conducted to examine the factorial validity of the Lithuanian version of CAPL-2. The four-domain model demonstrated a good fit to the data [*χ*^2^ = 106.26, df = 37, CFI = .963, TLI = .946, RMSEA = .047 (90% CI .037–.058), SRMR = .040]. The final model included an *a priori*-specified correlation between the residuals of the Predilection and Adequacy indicators. All standardized factor loadings were statistically significant (*p* < .001) and ranged from .409 to .855 (Figure 2). The Knowledge quiz items indicator had the lowest standardized factor loading (.409).

Correlations among latent factors were generally positive and statistically significant. The strongest association was observed between Motivation and Confidence and Physical Competence, whereas Knowledge and Understanding demonstrated comparatively weaker relationships with the remaining domains. Consistent with the domain-level correlation analysis, no statistically significant association was observed between Daily Behavior and Knowledge and Understanding.

### 3.3 Age and gender differences across CAPL-2 domains

To examine the construct validity of the instrument, additional analyses were conducted to compare CAPL-2 domain scores across age groups and between sexes. One-way analysis of variance indicated significant age-group differences among boys for the total CAPL-2 score (F = 6.97, *p* < .001, *η*^2^ = .053), Knowledge and Understanding (F = 11.87, *p* < .001, *η*^2^ = .084), Physical Competence (F = 8.12, *p* < .001, *η*^2^ = .055), and Daily Behavior (F = 3.47, *p* = .016, *η*^2^ = .025). Among girls, significant age-group differences were found for the total CAPL-2 score (F = 3.59, *p* = .014, *η*^2^ = .029), Knowledge and Understanding (F = 16.36, *p* < .001, *η*^2^ = .114), and Physical Competence (F = 10.19, *p* < .001, *η*^2^ = .068). No significant age-group differences were observed for Motivation and Confidence in either sex or for Daily Behavior among girls. Descriptive statistics by age and sex are presented in Table 2.

**Table 2:** CAPL-2 domain scores according to age and sex.

Measure	Sex	8 y	9 y	10 y	11 y
Total CAPL-2	Boys	67.9 ± 12.1	74.8 ± 12.6^8^†^^	73.3 ± 12.8^8^†^^	76.1 ± 7.2^†^
	Girls	65.1 ± 10.2	69.0 ± 11.6	69.5 ± 11.1^8^	67.1 ± 10.6
Motivation and Confidence	Boys	26.1 ± 4.1^†^	26.4 ± 4.0	26.0 ± 3.9	26.1 ± 3.5
	Girls	24.7 ± 4.8	25.7 ± 3.8	25.5 ± 3.9	24.4 ± 4.4
Knowledge and Understanding	Boys	4.4 ± 2.4	5.1 ± 2.3	6.0 ± 2.2^8^^,^^9^	6.4 ± 1.5^8^
	Girls	4.3 ± 2.0	5.4 ± 2.2^8^	6.1 ± 2.08^8^^,^^9^	6.5 ± 2.4^8^
Physical Competence	Boys	17.8 ± 6.1^†^	21.3 ± 5.6^8^†^^	20.6 ± 6.3^8^†^^	21.2 ± 6.2
	Girls	15.5 ± 4.9	18.0 ± 5.3^8^	18.6 ± 4.8^8^	19.3 ± 3.9^8^
Daily Behavior	Boys	17.7 ± 7.0	20.4 ± 6.8^8^†^^	18.8 ± 7.1	20.4 ± 6.9
	Girls	17.7 ± 6.6	18.0 ± 6.1	18.4 ± 6.0	16.4 ± 5.5

8, 9 : Significant difference from the age indicated by the respective number.

† Significant difference between sexes.

Within individual age groups, significant sex differences were observed in several CAPL-2 domains, with boys scoring higher than girls in: 8-year-old children–Motivation and Confidence [*t*(218) = 2.786, *p* = .006, Cohen's *d* = 0.38] and Physical Competence [*t*(216) = 2.908, *p* = .004, Cohen's *d* = 0.39]; 9 years–the total CAPL-2 score [*t*(239) = 3.693, *p* < .001, Cohen's *d* = 0.48], Daily Behavior [*t*(246) = 3.334, *p* < .001, Cohen's *d* = 0.42], and Physical Competence [*t*(248) = 4.650, *p* < .001, Cohen's *d* = 0.59]; 10 years–the total CAPL-2 score [*t*(261) = 2.578, *p* = .010, Cohen's *d* = 0.32] and Physical Competence [*t*(268) = 3.043, *p* = .003, Cohen's *d* = 0.37]; and 11 years–the total CAPL-2 score [*t*(31) = 2.856, *p* = .008, Cohen's *d* = 1.00] and Daily Behavior [*t*(31) = 2.765, *p* = .010, Cohen's *d* = 0.96]. Across all age groups, no significant sex differences were observed in the Knowledge and Understanding domain (Table 2).

### 3.4 Internal consistency

The internal consistency of the Motivation and Confidence domain was assessed using Cronbach's alpha coefficient and McDonald's omega. The 12-item scale demonstrated good internal consistency, with a Cronbach's alpha of .867 and a McDonald's omega of .87, indicating satisfactory reliability of the Lithuanian version of the instrument.

Corrected item–total correlations ranged from .399 to .665, suggesting adequate homogeneity across all items. Furthermore, the deletion of any individual item did not improve the overall reliability coefficient, with Cronbach's alpha values ranging from .850 to .867 when items were removed. These findings indicate that all items contributed meaningfully to the measurement of the Motivation and Confidence domain and support the retention of the original item structure.

## 4 Discussion

This study is the first cultural adaptation and psychometric evaluation of CAPL-2 among Lithuanian primary school children. The Lithuanian version of CAPL-2 demonstrated satisfactory structural validity and internal consistency for the Motivation and Confidence domain, supporting the applicability of the original four-domain model in children aged 8–11 years. Overall, the findings were broadly consistent with those reported in the original Canadian study (24) and subsequent international adaptations, supporting the cross-cultural applicability of the CAPL-2 framework (26–28, 30). For example, the Lithuanian version demonstrated a comparable model fit (CFI = 0.964, RMSEA = 0.045) to that reported for the original Canadian CAPL-2 model (CFI = 0.947, RMSEA = 0.041) (24), while retaining the original four-domain structure.

The model fit indices met recommended thresholds, supporting the applicability of the original CAPL-2 measurement model in the Lithuanian context. Importantly, all indicators were retained in the Lithuanian model, providing further support for the original CAPL-2 measurement framework. This finding is particularly noteworthy because previous adaptations have required model modifications to achieve acceptable fit. In the present study, the CFA model was specified following the approach adopted in the Danish validation, where correlations between the Predilection and Adequacy subscales within the Motivation and Confidence domain were permitted (28). The successful use of the same approach in the Lithuanian sample suggests that this relationship may not be unique to the Danish context. In contrast, the Chinese validation required the removal of one Knowledge and Understanding item and the Predilection indicator due to low factor loadings (27).

Beyond the psychometric evaluation, this study also provides the first PL profile of Lithuanian primary school children assessed using CAPL-2. The overall pattern of results was broadly consistent with findings reported in previous CAPL-2 studies. Across domains, Motivation and Confidence demonstrated the most favorable profile, reaching almost 86% of the maximum possible score. This finding is consistent with results reported in Greece (26) and China (27), suggesting that positive attitudes toward movement and PA may already be well established during the primary school years. From an educational perspective, these findings are encouraging, as motivation and confidence are considered important prerequisites for sustained participation in PA throughout life (13). Nevertheless, because the Motivation and Confidence domain relies on self-reported responses, the relatively high scores should be interpreted with some caution, as social desirability bias may lead children to report more favorable levels of motivation and confidence toward physical activity, although its influence appears to depend on the construct being measured (45, 46). Across all four CAPL-2 domains, Knowledge and Understanding showed the weakest correlations with the remaining domains and no significant association with Daily Behavior. This pattern is not unique to our sample: weak links between Knowledge and Understanding and the remaining domains have been reported in the original Canadian validation (24), in Denmark (28), in Pakistan (30), and in Greece, where children achieved high motivation and confidence scores alongside comparatively poor performance on knowledge items (26). The recurrence of this pattern across culturally and curricularly diverse samples suggests that it is unlikely to be a country-specific finding and may reflect characteristics of the construct and the way it is operationalized within CAPL-2.

Several non-exclusive explanations are plausible. Psychometrically, the Knowledge and Understanding domain is assessed with fewer items and a narrower 0–10 score range than the other domains (0–30), which may limit score variability and contribute to weaker observed correlations. Curricularly, as Dania et al. (26) noted, activity-oriented physical education practices do not necessarily translate into stronger cognitive outcomes related to PA and health; children can be motivated and active without acquiring the underlying conceptual knowledge targeted by the CAPL-2 items. In addition, the relatively high Motivation and Confidence scores observed across several validation studies may have reduced variability within this domain, potentially contributing to the modest associations with Knowledge and Understanding (26). Although the present study was not designed to evaluate the physical education curriculum directly, this pattern may reflect a greater emphasis within school-based physical education on participation in physical activity than on the explicit development of conceptual knowledge related to health, movement, and physical activity. If supported by future research, these findings could support greater integration of cognitive learning objectives into physical education practice.

Another finding of the present study was the presence of differences across age groups in several CAPL-2 domains. The most consistent age-related differences were observed for Knowledge and Understanding and Physical Competence. Scores increased steadily in both sexes (boys: 4.4–6.4; girls: 4.3–6.5), with no significant sex differences at any age. These findings were further supported by moderate age effects for the Knowledge and Understanding domain (*η*^2^ = 0.084 in boys and *η*^2^ = 0.114 in girls). This is the expected pattern for a cognitive domain that tracks formal schooling: declarative knowledge about PA and health accumulates with age and curricular exposure independently of sex. As for Physical Competence, both sex groups gained across the four-year span (boys: 17.8–21.2; girls: 15.5–19.3), but scores of boys were significantly higher at ages 8, 9, and 10. The gap narrowed by age 11, when the sex difference was no longer significant. These age-related changes were accompanied by small-to-moderate effect sizes (*η*^2^ = 0.055 for boys and *η*^2^ = 0.068 for girls), indicating meaningful developmental improvements in physical competence. This suggests that girls' motor-competence development closes part of the early advantage held by boys, though the absolute gap remains. In contrast, Motivation and Confidence remained relatively stable across age groups. This was reflected in very small age effects (*η*^2^ = 0.001 for boys and *η*^2^ = 0.012 for girls). The high relatively stable scores may reflect a ceiling effect in this domain (with a mean around 25–26 out of 30) rather than genuine developmental stability, and it limits the domain's ability to discriminate change over this age range. Comparable trends have been reported in Greece (26) and Pakistan (30), where overall PL, physical competence, and knowledge and understanding scores increased with age. Together, these findings suggest that older children may have had more opportunities to develop movement-related competencies and acquire knowledge relevant to PA and health. While Daily Behavior showed age-related differences only among boys. Scores of boys stayed around 18–20 at all ages, with a peak at age 9 that produced the only significant sex difference. Scores of girls were broadly stable from 8 to 10 years (17.7 to 18.4) but dropped to 16.4 at age 11. Consistent with these findings, age effects for Daily Behavior were small in boys (*η*^2^ = 0.025) and negligible in girls (*η*^2^ = 0.005). Although this decline was not flagged as statistically significant in pairwise terms, it is directionally consistent with the international evidence that habitual PA begins to decrease in girls during the transition to early adolescence and warrants attention as a target for intervention (47, 48).

From a theoretical perspective, the present findings provide further support for Whitehead's conceptualization of physical literacy as a holistic construct comprising interconnected yet distinct domains that develop throughout childhood (12, 13). Although these domains are conceptually related, they do not necessarily follow identical developmental trajectories. In the present study, Knowledge and Understanding and Physical Competence demonstrated clear age-related improvements, whereas Motivation and Confidence remained relatively stable and Daily Behavior showed only limited age-related variation. These findings reinforce the multidimensional nature of physical literacy and support the multidomain approach to its assessment adopted by CAPL-2.

From a practical perspective, the availability of a culturally adapted and validated Lithuanian version of CAPL-2 provides the first standardized tool for assessing physical literacy in Lithuanian primary school children. This study represents an important initial step towards the systematic assessment of physical literacy in Lithuania and establishes a foundation for future research. Additional studies are needed to generate national reference data, further evaluate measurement properties, and explore the practical application of CAPL-2 scores in educational and public health settings. Nevertheless, the Lithuanian CAPL-2 offers researchers, educators, and policymakers a common framework for assessing physical literacy and contributes to the growing recognition of physical literacy as an important component of child health and education in Lithuania.

This study has several important strengths. To our knowledge, it is the first study to translate, culturally adapt, and validate the CAPL-2 for use among Lithuanian children. The study was based on a large sample of 846 primary school children and employed a geographically stratified cluster sampling approach that included participants from all ten administrative regions of Lithuania. This sampling strategy ensured broad geographic coverage and captured the diversity of Lithuanian primary school settings, supporting the generalizability of the findings. Another strength is the rigorous translation and cultural adaptation process, which followed established international recommendations and included forward translation, back-translation, expert review, and pilot testing. In addition, all assessments were conducted by the same trained research team using standardized testing procedures, helping to ensure consistency in data collection across study sites. Together, these methodological procedures contributed to the linguistic, cultural, and measurement quality of the Lithuanian version of CAPL-2.

Several limitations should also be acknowledged. First, due to incomplete data for some CAPL-2 assessment components, the number of valid observations varied across analyses. Nevertheless, the remaining sample sizes were large and considered adequate for psychometric evaluation. Second, although internal consistency and construct validity were evaluated, test–retest reliability was not assessed because the study employed a cross-sectional design and data were collected at a single time point. Consequently, the temporal stability of the Lithuanian CAPL-2 remains to be established and should be examined in future studies. Third, convergent and criterion validity were not evaluated against external measures. Future research should therefore examine the relationships between the Lithuanian CAPL-2 and other established indicators of physical activity, motor competence, and health outcomes. Fourth, the cognitive debriefing was conducted with a relatively small sample of six children. Although this sample size is consistent with commonly used procedures in cross-cultural adaptation studies, a larger and more diverse sample might have identified additional issues related to item interpretation or cultural relevance. Fifth, daily step counts were obtained using a wrist-worn accelerometer rather than the waist-worn pedometer recommended in the original CAPL-2 protocol. Although standard CAPL-2 scoring procedures were applied, direct comparisons with studies strictly following the original protocol should be interpreted with caution. Finally, although comparisons across age groups provided additional support for the construct validity of the Lithuanian CAPL-2, the cross-sectional design did not permit conclusions regarding individual developmental trajectories. Longitudinal studies are needed to examine changes in PL over time.

## Conclusion

The Lithuanian version of CAPL-2 demonstrated satisfactory psychometric properties and retained the original four-domain structure in a large sample of Lithuanian primary school children. Despite the limitations, the findings support the cross-cultural adaptation, construct validity, and internal consistency of the Lithuanian version of CAPL-2, providing evidence that it is an appropriate instrument for assessing physical literacy among Lithuanian primary school children. The Lithuanian CAPL-2 may therefore serve as a useful tool for assessing PL in research, educational, and public health settings while enabling future national and international comparisons of children's PL.

## Data Availability

The raw data supporting the conclusions of this article will be made available by the authors, without undue reservation.
